# Tell Me What You Eat and I Will Tell You Your Sociotype: Coping with Diabesity

**DOI:** 10.5041/RMMJ.10077

**Published:** 2012-04-30

**Authors:** Elliot M. Berry, Sabina De Geest

**Affiliations:** 1 Braun School of Public Health, Faculty of Medicine, Hebrew University of Jerusalem, Israel;; 2 Institute of Nursing Science, Faculty of Medicine, University of Basel, Switzerland

**Keywords:** Coping, diabetes, environment, genotype, obesity, phenotype, relationships, sociotype

## Abstract

The term *sociotype* has been introduced to describe the dynamic relationship of an individual with his/her social environment throughout life. The sociotype is a conceptual framework to highlight, in addition to bio-medical pathways, the psycho-social and environmental factors necessary to understand responses to life stresses and patient self-management for chronic illness. The sociotype interacts with genotype expression through mate selection and metabolic programming, and with the phenotype to determine adaptation throughout life from birth to old age. Following on the work of Antonovsky, Engel, and McEwen, and others in the life and social sciences, the sociotype details and expands the many factors generally included in the environmental influences on a person’s life identified here as the domains of *health*, *relationships*, and *environment*. Physiological mediators for sociotypic influences include: adrenal steroids and the sympathetic nervous system (allostatic load), and oxytocin (social neuroscience). The biological pathways are multiple through nutrition (essential dietary-derived amino- and fatty acids for neurotransmitter synthesis, caloric restriction, and diet–gene interactions), epigenesis, and metabolic programming. Nutrition influences growth and development, fertility and longevity, and also determines susceptibility to non-communicable diseases such as cardiovascular disease and cancer, and particularly diabetes and obesity, through *in-utero* effects, the development of intestinal flora (microbiome), and chronic stress. Thus the sociotype and nutrition are reciprocally related in both health and disease.

It is not the consciousness of men that determines their existence, but, on the contrary, it is their social existence that determines their consciousness.

(Preface to the *Critique of Political Economy*, Karl Marx, 1859)

## INTRODUCING THE SOCIOTYPE

**Figure 1:**
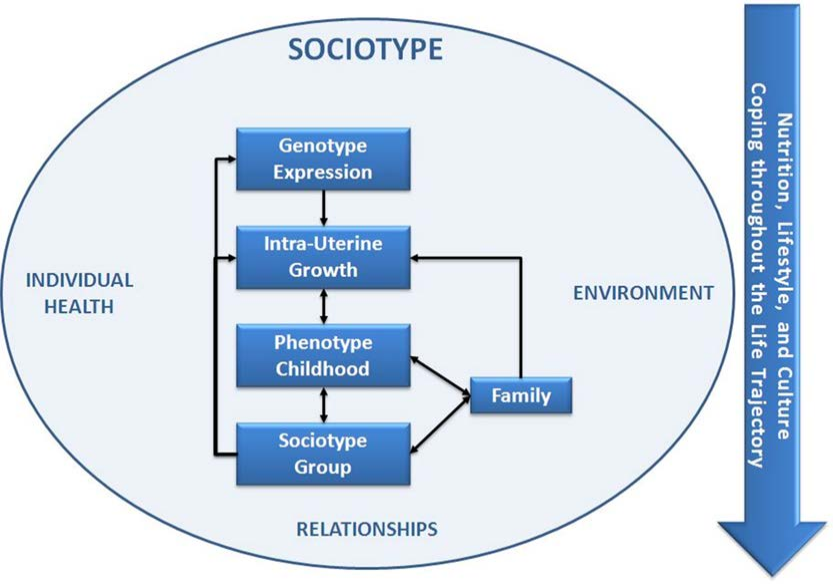
**The relationship of the sociotype to genotypic expression and the phenotype throughout the life cycle.**

The term *sociotype* has been introduced to describe the dynamic relationship of an individual with his/her social environment throughout the life trajectory.^1^ It is a framework for understanding how people manage life in general, and chronic disease in particular. The sociotype interacts with genotype expression through, for example, mate selection, epigenesis, and metabolic programming, and with the phenotype throughout life from birth to old age. The sociotype is an explanatory framework that analyses and expands the many factors usually included in the *environmental* influences on a person’s life. The sociotype is constituted by *individual health*, *relationships*, and *environment.* Every person is thus a product of the prevailing mores and his/her “three-fold cord”—genotype, phenotype, and sociotype. Figure 1 shows these interactions.

## ORIGINS OF THE SOCIOTYPE

The sociotype framework embraces many previous insights that have led to theoretical and derived concepts that position human functioning throughout life, health, and illness in a broader bio-psycho-social framework^2^ and describe how people cope with life circumstances.^3^,^4^ Therefore, the sociotype is a synthesis of the ideas of many scientists and health professionals from the disciplines of physiology, psychology, medicine, nursing, sociology, and anthropology. Major intellectual debts are acknowledged to the following thinkers and scientists who were interested in understanding the human situation. In alphabetical order they include: Adler,^5^ Bandura,^6^ Berkman,^7^ Bowlby,^8^ Erikson,^9^ Frankl,^10^ Freud,^11^ Fromm,^12^ Greenfield,^13^ Harlow et al.,^14^ Horney,^15^ Jung,^16^ Levi-Strauss,^17^ Lorenz,^18^ Marx,^19^ Maslow,^20^ Piaget,^21^ Rogers,^22^ Seligman,^23^ and Winnicott^24^. Even this list is not exhaustive but indicates how long-standing were (and are) such attempts to formulate theories and coping that go beyond bio-medical factors. This approach may be summarized by the well-known saying of Sir William Osler (1849–1919): “It is more important to know what kind of patient has the disease than what kind of disease the patient has.” From this developed the bio-psycho-social model of disease of Engel, which gave a *static* definition of the origins of disease rather than health and was less concerned with life-history *dynamics*. Antonovsky pioneered the concept of healthiness or salutogenesis, and positioned a person on a continuum between health and disease depending on the balance between risks and resources.^3^,^25^ He postulated the importance of a “sense of coherence” recognizing three areas—personal, social, and ecological.

Following his scheme, in the sociotypic model these have been defined as the domains of *individual health*, *relationships*, and *environment*, respectively (Table 1). We define “successful” sociotypic development as a mature, healthy, independent individual functioning in, and contributing to, society according to his/her full potential. The sociotype is culture-dependent, and therefore there is no comprehensive definition of a “normal” sociotype.

**Table 1:** **The three domains of sociotypic inputs throughout the life trajectory.** Classification of some of the factors that are involved in dealing with different life situations. This Table should be read horizontally: some factors may operate and change at more than one life-time stage.

Timeline	Individual Health	Relationships with...	Environment
*In utero*	Nutrition Growth Development	Type of family (one or two parents, same sex)	Geographic demography Urban or rural Environmental exposure Migration
Infancy and Childhood	Breast-feeding Physical, psychological, and emotional health Special needs Personality Activity Sleep pattern	Parents (including their relationship) Siblings (birth order), adoption, grandparents, cousins, Pets	Education, stimulation Indoctrination: Religious, political Realizing potential Screen time (computer, TV)
Adolescence	Rites of passage Health behaviors, including sexuality Substance use, including: tobacco, alcohol, drugs, eating behavior	School friends, peer group Social networks	Higher education Vocational training Military service
Adulthood	Personality type Sense of humor Creativity Separation and individuation Spiritual/religious–existential belief system(s) Self-knowledge Shadows	Spouse (mate selection) and in-laws Children (letting go) At work: Superiors and colleagues Lovers Sexual activity	Employment, occupation, career trajectory Home, leisure time, music, art, literature Media and information technology Socio-economic class, religious values
Middle Age	Coping strategies through life events, including: Marriage, birth, infertility, divorce, menopause, retirement, disease, bereavement, misfortune, bankruptcy	Care of parents Grandchildren Friends Strangers, herd behavior	Political system and ideology National Identity Social class, societal values, social justice Culture Disasters: Natural, man-made
Old Age	Physiological, psychological, and cognitive decline Chronic disease Chronic pain Thoughts of mortality	Family Friends Helpers	Housing, sheltered living, institution

The following are examples of some of the questions that the sociotype attempts to address in the three domains:

*Health:* How to live with special needs? How to cope with living following a stroke, with inflammatory bowel disease, rheumatoid arthritis, or post-transplant? How do Holocaust survivors survive?

*Relationships*: What determines how we choose our mates/partners? How to deal with divorce or bereavement?

*Environment*: How to deal with bankruptcy, job dismissal, retirement? How to deal with wars and their aftermath?

Some of the above overlap domains in their effects—consider the consequences of a serious road traffic accident. The answers are not given only by quantitative methodologies but require a combination of qualitative and quantitative methodologies (mixed methods).

The thrust of this paper is to explain the sociotype framework in more detail to understand the multiple interrelations of its components necessary for functioning in health and illness. We will first describe the different aspects of the three domains and then indicate the crucial importance of nutrition on sociotypic development from pregnancy to old age in health and in relation to the development of diabesity.

## CONCEPTUALIZING THE SOCIOTYPE: THE THREE DOMAINS—INDIVIDUAL HEALTH, RELATIONSHIPS AND ENVIRONMENT

Table 1 is an attempt to arrange the sociotypic factors acting at different times during the life cycle as inputs in the three domains. It is not exhaustive and varies with the individual’s location and living conditions. The entries vary widely as to importance or influence in any given individual, although some attempt has been made to give a hierarchical structure.

In the *health* domain there is the importance of accrued life experiences, beginning with bonding and imprinting, influencing personality development and even a sense of humor. These develop slowly in human maturation as the period of infancy, childhood dependency, and adolescence has increased during hominid evolution, as well described by Hochberg^26^ and Konner.^27^ Secure or insecure infantile and childhood parental attachment is considered to program reproductive strategies.^28^ Sleeping, sexuality, and eating (the dark side of the moon) occupy a third of a person’s life and are most relevant to his/her equilibrium, yet are rarely discussed in most clinical case descriptions.

The domain of *relationships* considers those of family, peer group and friends, lovers, and people in authority. Mate selection is a key example of the reciprocal interaction between the phenotype and the sociotype, determining the genetic make-up of the next generation by shuffling the gene pool—but it is yet to be determined how much is biologically or psychologically driven.^29^,^30^ It is suggested that humans select major histocompatibility complex (MHC)-dissimilar partners through olfactory (pheromone) and other cues so as to enhance offspring heterozygosity; the effect of perfumes, cosmetics, and deodorants on this is a major concern.^31^ Such biological mechanisms are examples of the effect of the phenotype on the sociotype. Marriage avoidance among their peer group by kibbutz children is clearly due to sociotypic influences.^32^ Dealing with parental approval can be a lifelong task. Communication is at a number of levels—intellectual, emotional, spiritual, and physical.

Factors covered by the *environment* domain include education, employment, economic circumstances, and time spent at work, home, and during leisure activities. Political, ideological, and societal values influence behavior. Most people appear to be Marxist *within* the family, yet Capitalist *outside* of it.^33^ The effects of disasters, whether natural (tsunamis, earthquakes) or man-made (wars, economic crises), have long-term effects on the sociotype. The recent economic crisis in Greece has already had health consequences.^34^,^35^ Other factors that have a significant influence on health behaviors are social networks, media, and the information technology revolution.^36^ Religiosity spans all domains in attempting to provide a meaning to one’s situation as well as communal support,^37^ while others find in humanism an alternative answer to such needs. There may be counter-pressures relating to individuality and creativity.

The position of elements in Table 1 is open to debate. For instance, societal values found under *environment* could also be placed in *health* or *relationships*, which only shows that these factors are not easily categorized. Yet this is not a problem; rather, the three domains may be likened to the palate of the three primary colors that shade the many different influences of, and responses to, the sociotype as has been shown for the sense of coherence scale.^38^ Thus, there may be intermediate groupings and cross-classifications among the domains. For example, *health–relationships* include maternal bonding and attachment, community and family support systems. *Environment–relationships* express socio-economic conditions and work opportunities. National identity factors are examples of cross-boundary issues as between French and Flemish speakers (in Belgium), Rumanians and Moldovans, Israeli and Palestinian Arabs, North and South Irish, and elsewhere. The *health–environment* axis would include the physical environment and air pollution as well as access to health systems and treatment. Catastrophes such as a war or the global economic situation affect all three domains.

Sociotypic factors work at more than one stage in life (e.g. physical handicap, existential doubts, or a chronic disease). For example, natural or man-made disasters and disease may occur at any time; spiritual or ideological beliefs and taste in music may also change with age and maturity. It is thus obvious that the various influences on the sociotype may operate and change *at different times* and *to different extents* throughout the life cycle. Education is not just from childhood to university but has far-reaching effects throughout life. In different societies other factors may be relevant such as cultural acceptance of disease, health literacy, and the impact of social networks. And overall, there is the influence of chance and the realization that life events cannot be easily predicted or classified. There may even be a danger that the sociotype encompasses so many variables that, in the words of the Talmudic dictum, “If you grasp a lot you cannot hold it, if you grasp a little you can hold it” (Babylonian Talmud tractate Rosh Hashana 4b). Thus, the challenge is to find the specific factors operating for any given person in the particular life situation.

## BIOLOGICAL PATHWAYS FOR SOCIOTYPIC INFLUENCE

**Figure 2:**
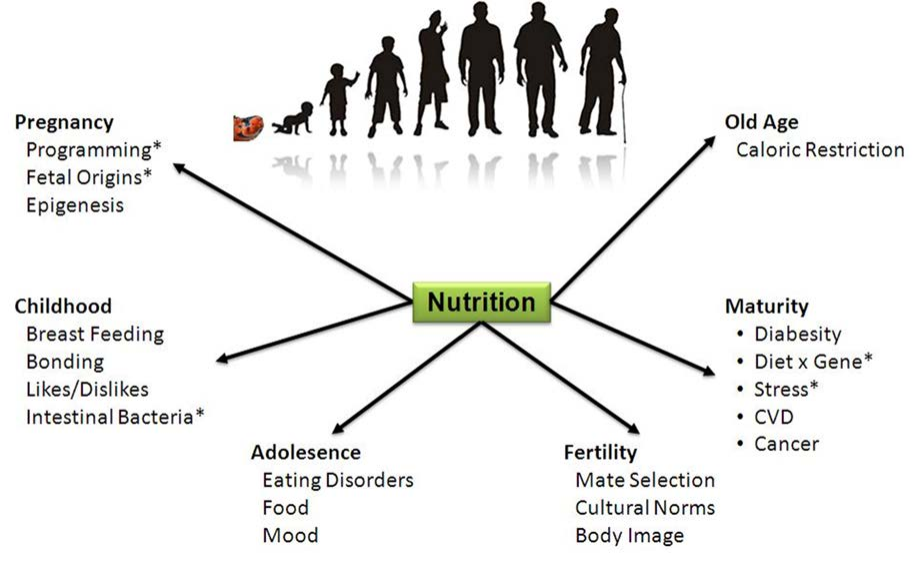
**The effects of nutrition on the sociotype during the life cycle.** ^*^Factors involved in the pathogenesis of diabesity as discussed in the text.

The idea of the sociotype would be of little value if there were no biological pathways through which it could influence health and functioning. It provides a bridge between individual health and public health.^7^,^39^ The concepts of allostasis and allostatic load have been championed by McEwen to describe how chronic stress—”wear and tear”—can affect well-being through over-action of adrenal steroid secretion and the sympathetic nervous system.^4^,^40^ Oxytocin and vasopressin secretion are other pathways with multiple effects on birth, lactation, bonding, mate selection, and aggression. There are gender differences in the sociotypic responses, with females tending to *(de)fend and befriend* and males to *fight or flight*.^41^ This has become the basis for the new discipline of social neuroscience. We believe, however, that one of the major pathways of influence on sociotypic development during the human life cycle is through nutrition, and this is shown in Figure 2.

## THE ROLE OF NUTRITION ON THE SOCIOTYPE DURING THE LIFE CYCLE

Nutrition may be likened to the fuel that drives the body’s physical and psychological motor and should therefore be of the highest octane. From the research on the neonatal origins of adult disease (Barker hypothesis and metabolic programming)^42^ and longevity (telomere lengths^43^–^45^) it is now recognized that nutritional influences begin at minus nine months. Famine pregnancy studies from Holland^46^ and China^47^ confirm the increase in schizophrenia in the adult progeny. Nutrition affects many stages: pregnancy, breast-feeding, and weaning. The concept of the bonding and the “good and bad breast” stresses the importance of parenting attitudes. Milk contains endocannabinoids which are crucial for infant suckling.^48^ The endocannabinoid neurotransmitter modulator system is derived from essential omega-6 polyunsaturated fatty acids necessary for the synthesis of 2 arachidonyl glycerol and regulates appetite in a quasi-feedback loop.^49^ In rodents, maternal grooming influences serotonin activity, steroid secretion, and the methylation and acetylation status of gene expression.^50^

Adolescents are vulnerable to cultural influences concerning body image and to the development of eating disorders. In some people nutrition may affect their cognitive assessments (food and mood).^51^ Three of the principal neurotransmitters—serotonin, norepinephrine, and dopamine—are derived from essential amino-acids tryptophan, phenylalanine, and tyrosine. It is possible that some of the abnormal cognitive functioning in anorexia is due a lack of such precursors, which can be alleviated by oral tyrosine supplementation (Israel, Avraham & Berry, unpublished data). Eating disorders are examples par excellence of culture-bound diseases of the sociotype (“nothing tastes as good as thinness feels”), as discussed elsewhere.^1^ Brain peptides—orexins—provide a link between eating behavior, body weight, and sleep.^52^,^53^

Fertility: The age of the menarche is dependent on the amount of energy stores (adipose tissue) required to sustain a pregnancy^54^ and has become earlier in most countries^55^ as nutrition has improved; conversely, amenorrhea is a (necessary) consequence of anorexia.

Maturity: Discussion of the many examples of *diet–gene* interactions^56^ is beyond the scope of this review but has obvious relevance to the sociotype. It has been said that a family who eats together, sticks together.

Old age: Caloric restriction is the one proven means to increase longevity and involves *inter alia* sirtuin pathways.^57^ Leptin, which is secreted in proportion to adipose mass, has multiple functions. We have shown it to be involved in survival under conditions of severe caloric deprivation^58^ as well as being neuro-protective.^59^ Indeed, recent studies suggest that survival of severely ill intensive care patients is associated with higher levels of leptin (Sviri, Avraham, Berry, et al., submitted). Of interest is that omega-3 essential dietary fatty acids may exhibit some actions similar to those of leptin regarding survival and cognitive function.^60^

Finally, nutrition is also involved in resistance to infectious diseases through effects on the immune system and intestinal bacteria.^61^ The sociotype influences involve public health measures concerning sanitation and immunization policies.

## THE PROBLEM OF DIABESITY

The role of nutrition in the pathogenesis of non-communicable diseases such as diabesity, cardiovascular disease, and cancer are well known.^62^ There are at least three pathways whereby nutritional status can lead to the development of diabesity: (1) impaired fetal growth, (2) intestinal bacteria, and (3) increased allostatic load of chronic stress (Figure 2).^40^ If a pregnant woman is stressed or malnourished, the fetus’ development may be affected, leading to increased risk of insulin dependent diabetes mellitus (NIDDM), cardiovascular disease, and hypertension in later life.^42^ Intestinal flora can regulate insulin sensitivity through a number of pathways involving fatty acid oxidation, inflammatory lipopolysaccharides (LPS), short chain fatty acid lipogenesis, incretin secretion, and butyrate production.^61^ It remains to be determined how diet regulates the colonization of intestinal bacteria (the microbiome) and how they may, in turn, influence energy balance.^63^ Finally, metabolic efficiency increases with age, leading to the relentless weight increase observed in developing countries over the decades of life.

The sociotypic effects of the environment are clearly demonstrated by comparing the normal to mild overweight of Pima Indians living *traditionally* in Mexico to the severe obesity among their fellow tribe members and relatives living *affluently* in Arizona on reservations, some running casinos.^64^ Thus the pandemic of obesity may be considered a *normal* response to an *abnormal* environment that encourages too much eating and too little activity. In industrialized countries obesity is inversely related to socio-economic status and years of education. That the opposite occurs in low-income countries shows the influence of socio-economic and cultural norms.^65^ Of course there is a genetic component to obesity and metabolic efficiency, which was an advantage during evolution when food sources were inconstant.^66^ However, in recent times this has turned into a handicap. Obesity, therefore, is inherited but not inevitable, and cannot occur unless there is a permissive “obesogenic” environment.^67^

The effects of social networks on the sociotype have been described for obesity,^68^ and recent research has shown the benefits of moving house on the prevalence of diabetes and obesity.^69^ This would not seem to be a universally available option, and the mechanisms are not clear, though presumably they involve changes in life-style influenced by the new neighborhood.

## THE SOCIOTYPE OF DIABESITY AND CHRONIC ILLNESS: PATIENT SELF-MANAGEMENT

Table 2 lists aspects of the sociotype in the three domains—in *addition* to the factors detailed in Table 1—that are required for coping with chronic disease, using diabesity as an example. The sociotypic map will change during the course of an illness. For instance, the response to a relapse following cancer therapy or in multiple sclerosis will not be the same as that at the initial diagnosis. And in a diabetic patient, the need for dialysis or an amputation will produce a different sociotypic response than that for commencing injections of insulin.

**Table 2:** **Additional factors in the three domains of the sociotype that relate specifically to chronic disease management as in diabesity.**

**Individual Health**	**Relationships**	**Environment**
Macro-level: Health care system, health benefits	Significant other and family	Access to health centers, mobility
Meso-level: Health insurance, relations with health maintenance organization (HMO), pharmacists	Community primary care case manager and medical team	Facilities for chronic care: clinics, dialysis centers, day-care
Micro-level: Patient care process, health literacy, treatment, and side-effects: adapting, adjusting to a new reality	Case manager Hospital specialist Medical team and health professionals	Financial circumstances
Physical status, nutrition, activity, self-management	Continuity and coordination of care	Housing facilities (daily living) Access to care, personal hygiene facilities, etc.
Mental health, personality type, health beliefs, taking responsibility for treatment options	Patient self-management support groups	Ability to continue working
Coping and compliance strategies, denial Dependency, loss of control Complications and side-effects Eyesight Peripheral vascular disease (amputations) Kidneys (dialysis) Impotence	Friends Pets Carers	Cultural acceptability of illness
Health literacy Alternative medicine		Information technology

The key factors integral to the management of diabesity are a change in diet and life-style,^70^ and encouraging self-management,^71^,^72^ using a combination of techniques of which motivational interviewing by health professionals is one example.^73^ Further, investments need to be made in strengthening competencies of the health team and implementation of new care models for a multidimensional approach to patient management. This involves new relationships with the case manager, hospital specialists, and carers.

Self-management also means that patients have the confidence to follow their prescribed therapy, to avoid health deterioration, and to preserve function.^74^,^75^ It is hypothesized that the sociotype is essential for the ability to succeed in the three self-management tasks of: (1) medical management, (2) role management, and (3) emotional management. For diabetes patients, the first task involves the skills needed in leading a healthy life-style and following correctly the medication regimen. In addition, the patient has to deal with the possible side-effects of the treatment as well as disease progression (macro- and micro-angiopathy). Role management refers to the managing of relationships that change or come under pressure during the course of the chronic illness. The third task of emotional management refers to the skills patients need to cope with emotional states or challenges associated with their illness.^74^

Self-management support consists of a portfolio of techniques and tools required to achieve these tasks, which calls for a fundamental transformation of the patient–caregiver relationship into a collaborative partnership.^75^ One practical way is to draw on the experience of patients from similar backgrounds who are already successful in managing their disease using, for example, the methodologies of positive deviance.^76^,^77^

The impact of the disease on the family cannot be overestimated in coping with new roles, and there may be the problem of impotence affecting sexuality. Obviously there are financial issues, especially if the patient is poorly insured and unable to continue his/her work.

Evidence from chronically ill populations, including diabetes, shows that improved outcomes occur when care systems shift from acute to chronic care paradigms, particularly if they include support for patient self-management.^78^–^84^ The new approach requires moving from predominantly acute-care-driven management plans which generally ignore behavioral, psycho-social, and environmental factors, towards models that guarantee effective long-term illness care. These should combine the following features: (1) provide comprehensive, multidisciplinary care, (2) integrate and co-ordinate care along the care continuum, (3) be disease or population-specific, (4) include tools to promote patient self-management, (5) be evidence-based, and (6) imbed information technology.^78^,^80^,^85^,^86^

There is also good evidence that adherence improves diabetic control^87^ thereby delaying complications.^88^,^89^ The challenge for health teams lies in promoting this goal, the economic consequences of which are obvious.^90^,^91^ Given that clinical results depend principally on patients’ daily self-management, tackling non-medical risk factors through interventions to support it sociotypically represents a potentially powerful pathway to improve long-term outcomes in the chronically ill.

## MANAGING THE PATIENT WITH CHRONIC DISEASE

For any given patient, the ability to cope with chronic disease is dependent on elements in the three sociotypic domains which, in turn and to varying degrees, determine the long-term outcome. The lists of factors in Tables 1 and 2 suggest that conventional medical education does not yet prepare future practitioners for such a task. It is difficult to envisage all the skills required since they are multidisciplinary, involving, in addition to medicine, the integration of the sciences of sociology, psychology, and anthropology *inter alia.* Systems biology has been proposed as the new direction, but the initial versions of it are still too bio-reductionist to encompass the necessary sociotypic elements.^1^,^92^,^93^

In order to assess patients with chronic diseases, the medical history has to be expanded to include consideration of the sociotype. This is much more than the conventional social history of living conditions and socio-economic circumstances. Furthermore, mental and physical health interact such that coping strategies may have “trade-offs” between them. A recent study indicates that they must be considered jointly when researching the causes of disparities across racial groups and questions implicit assumptions concerning associations between social disadvantages, health behaviors and mental health.^94^ A sociotypic history is an attempt to understand the totality of developmental experience—”to step into the patient’s shoes”—that determines reactions, responses, and obstacles to self-management.

## FOR THE FUTURE

It is clear that many of the factors detailed in Tables 1 and 2 do not lend themselves readily to quantification, especially with regard to relationships, and it may not be possible to give a global sociotype score. The point is that for any given patient there will be relevant issues that have to be assessed separately in each of the three domains. Future projects will identify both biomarkers^40^ and questionnaires that are relevant to the challenge of typing the sociotype (as examples: for family function, family Apgar score,^95^ stress,^96^,^97^ quality of life,^98^ and social adaptability^99^). This means that in its *strong* form sociotypic analysis will require establishing rigorous methods to investigate and quantify its effects.^40^,^75^ In its *weak* form sociotypic analysis is a way to highlight those psycho-social and environmental factors relevant to health and illness. Both approaches should prove to be a robust way of broadening health professionals’ vision of realities in health care, which will then enable appropriate individual, community, or national interventions for disease prevention and chronic disease management. Such knowledge could then inform policy and practice to improve public health. The expectation is that the greater the consideration of the sociotype, the better will be the treatment outcome and the patient’s self-management. To test this hypothesis is the future challenge. The comprehensiveness of the sociotype requires that its analysis be approached in a graded manner; the role of nutrition in its development throughout life would seem to be a suitable first step.

## CONCLUSIONS

This paper has indicated how nutrition in its widest sense has a major influence on the development of the sociotype throughout the life cycle. This was anticipated at the *individual* level by Brillat-Savarin (1755–1826) in his well-known saying: “Tell me what you eat, and I will tell you what you are.” We may broaden this construct to the level of *relationships*: “Tell me how a family eats, and I will tell you how it functions.” And finally, the extension of sociotypic analysis and nutrition to the level of the *environment*: “Tell me how a nation eats, and I will tell you its values”—do, for example, children go to bed hungry? (Food security). The sociotype determines how an individual adjusts to life in general and disease in particular. Thus, there is justification in claiming “Tell me your sociotype, and I will tell you what you eat” (and vice versa), from which then follows “Tell me your sociotype, and I will tell you how you cope.”

One cannot do better for a conclusion than quote the words of Spinoza (1632–1677) in *Tractatus Theologico-Politicus*, bk. 1, pt. 4:

*Sedulo curavi, humanas actiones non ridere, non lugare, neque detestari, sed intelligere.* I have striven not to laugh at human actions, not to weep at them, nor to hate them, but to understand them.

Indeed, it is not our role to be judgmental, but rather to try to understand better the person (patient) to help him/her cope with the disease and develop the full potential of his/her unique sociotype.
